# Evaluation of the* In Vivo* and* In Vitro* Effects of Fructose on Respiratory Chain Complexes in Tissues of Young Rats

**DOI:** 10.1155/2015/312530

**Published:** 2015-12-06

**Authors:** Ernesto António Macongonde, Thais Ceresér Vilela, Giselli Scaini, Cinara Ludvig Gonçalves, Bruna Klippel Ferreira, Naithan Ludian Fernandes Costa, Marcos Roberto de Oliveira, Silvio Avila, Emilio Luiz Streck, Gustavo Costa Ferreira, Patrícia Fernanda Schuck

**Affiliations:** ^1^Unidade Acadêmica de Ciências da Saúde, Universidade do Extremo Sul Catarinense, 88806-000 Criciúma, SC, Brazil; ^2^Departamento de Química, Universidade Federal de Mato Grosso, 78060-900 Cuiabá, MT, Brazil; ^3^Instituto de Bioquímica Médica Leopoldo de Meis, Universidade Federal do Rio de Janeiro, 21941-902 Rio de Janeiro, RJ, Brazil

## Abstract

Hereditary fructose intolerance (HFI) is an autosomal-recessive disorder characterized by fructose and fructose-1-phosphate accumulation in tissues and biological fluids of patients. This disease results from a deficiency of aldolase B, which metabolizes fructose in the liver, kidney, and small intestine. We here investigated the effect of acute fructose administration on the activities of mitochondrial respiratory chain complexes, succinate dehydrogenase (SDH), and malate dehydrogenase (MDH) in cerebral cortex, liver, kidney, and skeletal muscle of male 30-day-old Wistar rats. The rats received subcutaneous injection of sodium chloride (0.9%; control group) or fructose solution (5 *μ*mol/g; treated group). One hour later, the animals were euthanized and the cerebral cortex, liver, kidney, and skeletal muscle were isolated and homogenized for the investigations. Acute fructose administration increased complex I-III activity in liver. On the other hand, decreased complexes II and II-III activities in skeletal muscle and MDH in kidney were found. Interestingly, none of these parameters were affected* in vitro*. Our present data indicate that fructose administration elicits impairment of mitochondrial energy metabolism, which may contribute to the pathogenesis of the HFI patients.

## 1. Introduction

Accumulation of fructose and fructose-1-phosphate in tissues and biological fluids of patients is the main biochemical characteristic of the hereditary fructose intolerance (HFI), an autosomal-recessive disorder with an average incidence of 1 : 40,000 newborns [1]. The disease arises from a deficiency of aldolase B (fructose 1,6-bisphosphate aldolase; EC 4.1.2.13), responsible for metabolizing fructose in the liver, kidney, and small intestine [2, 3]. The main clinical and biochemical presentation in affected patients includes neurological impairment, hypoglycemia, vomiting, jaundice, renal tubular dysfunction, liver failure, hepatomegaly, metabolic acidosis, seizures, coma, and eventually death [4–9].

Fructose is phosphorylated to fructose-1-phosphate by fructokinase (EC 2.7.1.4) with the consumption of ATP [10]. It is presumed that hypoglycemia following fructose ingestion is caused by depletion of inorganic phosphate, which results in the inhibition of glycogenolysis (at the glycogen phosphorylase level) and gluconeogenesis [11]. Toxicity of fructose after intravenous administration at high doses results in hyperuricemia, hyperlactatemia, and ultrastructural alterations in liver and intestinal cells [12–14]. Furthermore, a fructose-enriched diet accentuates the accumulation of diacylglycerols in liver through induction of lipogenesis, leading to hepatic insulin resistance, metabolic syndrome, and consequently reduction of glucose tolerance in rodents [10, 15–17]. Fructose was also shown to promote oxidative stress in cerebral cortex, liver, and heart of rats [18–21]. However, the specific mechanisms involved in fructose toxicity are still poorly known.

Therefore, considering that fructose is the main accumulating metabolite in HFI and the mechanisms behind fructose toxicity are virtually unknown, in the present study we aimed to investigate the effect of acute fructose administration on the activities of mitochondrial respiratory chain complexes I-III, II, II-III, and IV and of succinate dehydrogenase (SDH; EC 1.3.99.1) and malate dehydrogenase (MDH; EC 1.1.1.37) activities in homogenates from central and peripheral tissues of young rats. We therefore mimicked the main biochemical finding observed in HFI patients by injecting fructose systemically in these animals in the hope of contributing to a better understanding of the pathophysiology of HFI complications.

## 2. Material and Methods

### 2.1. Fructose Solution

For the* in vivo* studies, fructose was dissolved in vehicle (0.9% sodium chloride). For the* in vitro* experiments, fructose was dissolved in the specific buffer for each technique. Fructose solution was always prepared on the day of the experiments and its pH was adjusted to 7.4.

### 2.2. Animals

Thirty-day-old male Wistar rats obtained from the Central Animal House of Universidade do Extremo Sul Catarinense were used. Rats were kept with dams until weaning at 21 days of age. The animals had free access to water and to a standard commercial chow and were maintained on a 12 : 12 h light/dark cycle in an air-conditioned constant temperature (22 ± 1°C) colony room. The “Principles of Laboratory Animal Care” (NIH publication number 80-23, revised 1996) and the “EC Directive 86/609/EEC” were followed in all experiments. All efforts were made to minimize the number of animals used and their suffering. This study was approved by the Local Ethical Committee on Animal Use for Research under the protocol 076/2013-2.

### 2.3. Fructose Administration and Tissues Preparation

The animals were divided into two groups: control group, which received a single subcutaneous injection of vehicle (0.9% sodium chloride), and fructose group, which received a single subcutaneous injection of fructose (5 *μ*mol/g of body weight or 0.9 mg/g of body weight), according to [20, 22]. Animals submitted to this experimental model present fructose serum levels of approximately 3.05 *μ*mol/mL (0.55 mg/mL) [20].

One hour after the administration, the animals were euthanized by decapitation under anesthesia (ketamine plus xylazine), and the cerebral cortex, kidney, liver, and skeletal muscle were rapidly excised on a Petri dish placed on ice and isolated. Cerebrospinal fluid was also collected, as well as total peripheral blood in order to obtain the serum. The structures were weighed and homogenized with SETH buffer, pH 7.4 (250 mM sucrose, 2 mM EDTA, 10 mM Trizma Base, and 50 IU/mL heparin). The homogenates were centrifuged at 800 ×g for 10 min at 4°C, and the supernatants were kept at −70°C until the enzyme activity determination. The maximal period between homogenate preparation and enzyme analysis was always less than 5 days.

For the* in vitro* experiments, tissue homogenates from animals without previous manipulation or drug administration were incubated in the absence (control group) or in the presence of increasing fructose concentrations (1 and 5 mM) during 60 minutes at 37°C. After that, samples were immediately assayed for enzyme activities.

### 2.4. Cerebrospinal Fluid Glucose and Serum Triacylglycerol Levels

Cerebrospinal fluid glucose and serum triacylglycerol (TAG) levels were measured by using a commercial kit in serum samples from rats receiving fructose (Labtest, Lagoa Santa, MG, Brazil), according the instructions of the manufacturer.

### 2.5. Spectrophotometric Analysis of Respiratory Chain Complexes I-IV

The activities of succinate-2,6-dichloroindophenol- (DCIP-) oxidoreductase (complex II) and succinate:cytochrome *c* oxidoreductase (complex II-III) were determined in the homogenates according to Fischer and colleagues [23]. The activity of NADH:cytochrome *c* oxidoreductase (complex I-III) was assayed in the homogenates according to the method described by Schapira [24] and that of cytochrome *c* oxidase (complex IV) according to Rustin and colleagues [25]. The methods described to measure these activities were slightly modified, as described in detail in a previous report [26]. The activities of the respiratory chain complexes were calculated as nmol·min^−1^·mg protein^−1^.

### 2.6. SDH Activity

SDH activity was determined according to the method of Fischer and colleagues [23], by following the decrease in absorbance, due to the reduction of 2,6-dichloroindophenol (2,6-DCIP) using phenazine methosulfate at 600 nm.

### 2.7. MDH Activity

MDH activity was measured according to Kitto [27], by following the reduction of NADH absorbance at 340 nm.

### 2.8. Protein Quantification

Protein concentrations were measured by the method of Lowry and colleagues [28] using bovine serum albumin as standard.

### 2.9. Statistical Analyses

Results are presented as mean ± standard error of mean. Assays were performed in duplicate or triplicate and the mean or median was used for statistical analysis. Data from* in vitro* experiments were analysed using one-way analysis of variance (ANOVA) followed by the* post hoc* Duncan multiple range test when *F* was significant.* In vivo* experiments results were analysed by Student's *t*-test for independent samples. Differences between groups were rated as significant at *p* < 0.05. All analyses were carried out in an IBM-compatible PC computer using the Statistical Package for the Social Sciences (SPSS) software 16.0.

## 3. Results

We first investigated the influence of acute fructose administration on cerebrospinal fluid glucose and serum TAG levels. We observed that glucose levels were similar between groups. On the other hand, TAG levels were increased in the animals receiving acute fructose administration, as compared to control group (*p* < 0.001). The data is depicted in Table 1.

**Figure 1:**
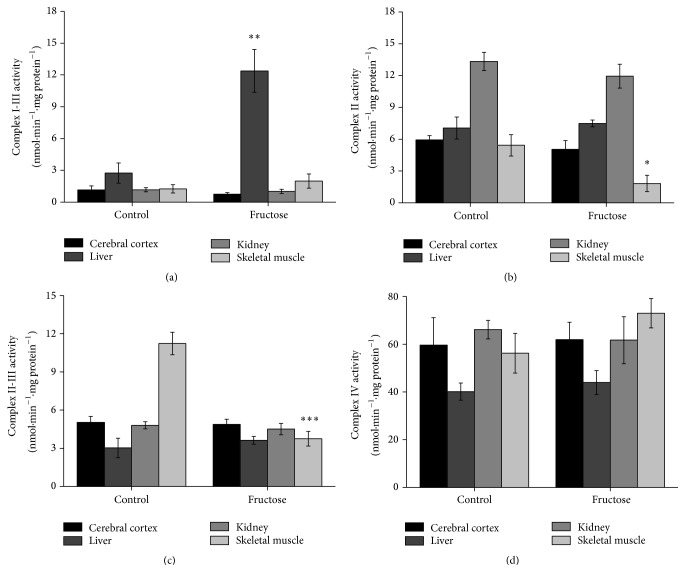
Effects of acute fructose administration on respiratory chain complexes I-III (a), II (b), II-III (c), and IV (d) activities in rat cerebral cortex, liver, kidney, and skeletal muscle. Values are means ± standard error of mean for five to six independent experiments performed in duplicate and are expressed as nmol·min^−1^·mg protein^−1^. ^*∗*^ *p* < 0.05, ^*∗∗*^ *p* < 0.01, and ^*∗∗∗*^ *p* < 0.001 compared to controls (Student's *t*-test).

We then investigated the influence of acute fructose administration on respiratory chain complexes activities. An increased activity of complex I-III in liver was observed (*p* < 0.01) (Figure 1(a)). We did not observe any alterations in respiratory chain complex I-III activity in the other studied tissues. On the other hand, a significant inhibition of complexes II and II-III activity was showed in skeletal muscle of animals receiving fructose acutely, as compared to control (*p* < 0.05 and *p* < 0.001, resp.) (Figures 1(b) and 1(c)) but not in the other tissues evaluated. Complex IV activity in the animals from fructose or control groups was also assessed. No differences were found between groups in cerebral cortex, kidney, liver, and skeletal muscle (Figure 1(d)). Furthermore, considering the effects of fructose administration on complex I-III in liver and complexes II and II-III in skeletal muscle, we investigated the* in vitro* effect of fructose (1 and 5 mM) on the same parameters. Fructose* in vitro* did not alter the activities of these complexes (Table 2).

**Figure 2:**
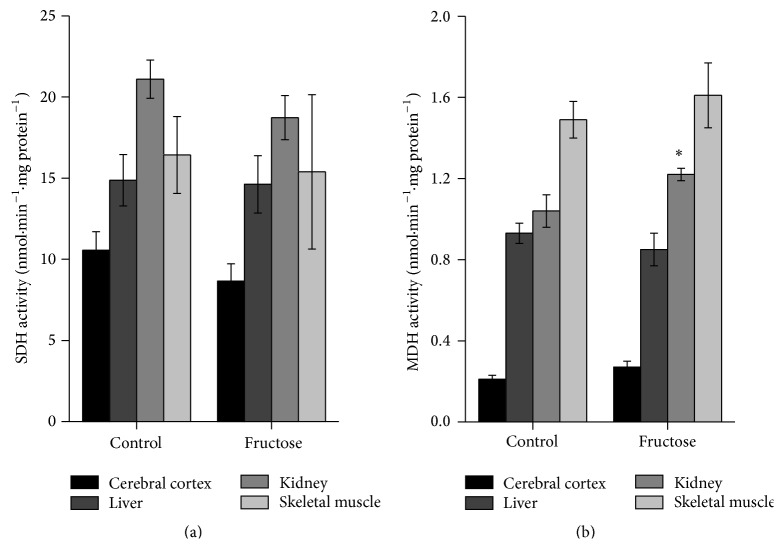
Effects of acute fructose administration on succinate dehydrogenase (SDH) (a) and malate dehydrogenase (MDH) (b) activities in rat cerebral cortex, liver, kidney, and skeletal muscle. Values are means ± standard error of mean for five to six independent experiments performed in duplicate and are expressed as nmol·min^−1^·mg protein^−1^. ^*∗*^ *p* < 0.05 compared to controls (Student's *t*-test).

Finally, we evaluated SDH and MDH activities in animals receiving fructose acutely. It was observed that MDH activity in kidney was increased as compared to control group (*p* < 0.05), while SDH activity was not affected in any tissue studied (Figure 2).

## 4. Discussion

HFI is characterized by a dramatic increase of fructose concentrations in tissues and biological fluids of patients [9]. In the present study, we demonstrated a dual effect on mitochondrial respiratory chain elicited by fructose administration in animals receiving this carbohydrate acutely. An increase of respiratory chain complex I-III activity in liver of rats and a decrease of respiratory chain complexes II and II-III activities in skeletal muscle were shown.

Inhibition of several enzymes of glycolysis and gluconeogenesis by an intracellular accumulation of fructose-1-phosphate is observed in HFI patients [2, 3, 29]. At least in part, such evidence suggests the putative modulation of these metabolic pathways as a mechanism that may help to substantiate the relative increase in the activity of complex I-III in liver of rats in the present work. Besides, mitochondrial respiratory chain complexes I and III are the main generators of mitochondrial reactive oxygen species (ROS) [30]. We have recently demonstrated that acute administration of fructose provokes oxidative stress in cerebral cortex of young rats [20] and other studies observed that high fructose diet might lead to oxidative stress in heart and liver of rats [18, 19, 21]. It has been also demonstrated that skeletal muscle cells exposed to fructose for up to 48 h presented oxidative stress, decreased mitochondrial DNA content, and mitochondrial dysfunction, which ultimately caused apoptosis by L6 myotubes [31]. In this context, ROS generated by mitochondria or from other sites within or outside the cell can cause damage to mitochondrial components and initiate degradative processes [24, 32–34]. Since fructose* in vitro* did not affect the respiratory chain complexes altered* in vivo*, a direct effect of this compound on respiratory chain is unlikely. Therefore, it cannot be ruled out that oxidative stress or other indirect stressors may be, in part, a contributing factor to the relative inhibition of mitochondrial complexes II and II-III in skeletal muscle of rats receiving fructose. Alternatively, the main fructose metabolite fructose-1-phosphate may also be accounted as a potential toxic metabolite in this experimental approach. Fructose administration also increased MDH activity in kidney, which is also an important mitochondrial enzyme involved in cell bioenergetics.

Defects of mitochondrial metabolism have a deleterious effect on cell function and survival, especially in highly energy-dependent tissues such as brain and skeletal muscle [35]. In the present study, the skeletal muscle showed a greater vulnerability to fructose administration on bioenergetics, as compared to the other tissues. Interestingly, Glut5 is mainly expressed in the liver [36] and it has a high fructose extraction rate [37], rendering liver accessible to virtually entirely ingested fructose. That may explain the dyslipidemia, hepatic steatosis, and insulin resistance elicited by fructose [10, 15, 17].

Some early studies suggested that consumption of a high fructose diet has harmful consequences for synaptic plasticity, impairs cognitive function, memory, dendritic spine density, and neurogenesis in the hippocampus, and induces neuronal loss [38–42]. Therefore, although we did not observe changes in the cerebral cortex in any of the parameters analysed, further studies in the various brain structures should be carried out before ruling out fructose neurotoxicity. In addition, Glut5 is only modestly expressed in nerve terminals therefore limiting the transport across plasma membrane of brain cells [43].

## 5. Conclusions

The results of the present study demonstrate that fructose disturbs mitochondrial energy metabolism particularly in peripheral tissues of rats. These findings are in line with the clinical features of HFI patients [4, 9, 28]. Mitochondrial dysfunction affecting ATP levels and availability can contribute to the onset of physiological abnormalities, as shown for the pathogenesis of several diseases [44]. Additionally, it has been recently shown that exposure to high fructose levels during gestation and lactation may induce long-term effects on mitochondrial function in aging rats [45] and also in our animal model [46]. It should be highlighted that chronic fructose exposition upregulates its own metabolic pathway [47], so that more studies evaluating high-sustained levels of fructose on these parameters would also be of great value. In conclusion, our present data indicate that fructose administration elicits impairment of mitochondrial energy metabolism, which may contribute to the pathogenesis of the HFI patients.

## Figures and Tables

**Table 1:** Glucose and triacylglycerol levels in CSF and serum of animals submitted to an animal model of fructosemia.

	Control	Fructose
Glucose (CSF)	70.6 ± 9.1	75.4 ± 8.7
Triacylglycerol (serum)	125.8 ± 16.9	252.6 ± 15.4^*∗*^

Values are mean ± standard error of mean for five independent experiments (animals) per group. Data were expressed as mg/dL. ^*∗*^ *p* < 0.05 compared to control (Student's *t*-test).

**Table 2:** *In vitro *effect of fructose on respiratory chain complex I-III in liver and complexes II and II-III in skeletal muscle of rats.

	Control	Fructose 1 mM	Fructose 5 mM
Complex I-III (liver)	4.17 ± 0.62	5.42 ± 0.38	4.05 ± 0.54
Complex II (skeletal muscle)	7.86 ± 0.26	7.82 ± 0.36	6.4 ± 0.85
Complex II-III (skeletal muscle)	4.56 ± 0.48	3.72 ± 0.63	3.31 ± 0.77

Values are mean ± standard error of mean for six independent experiments (animals) per group measured in the presence or absence of fructose. Data were expressed as nmol·min^−1^·mg protein^−1^. No significant differences were detected between groups (ANOVA).
